# MiR-203 down-regulates Rap1A and suppresses cell proliferation, adhesion and invasion in prostate cancer

**DOI:** 10.1186/s13046-015-0125-x

**Published:** 2015-01-31

**Authors:** Jun Xiang, Cuidong Bian, Hao Wang, Shengsong Huang, Denglong Wu

**Affiliations:** Department of Urology, Tongji Hospital, Tongji University School of Medicine, NO 389 Xinchun road, Shanghai, 200065 China; The Experimental Center of Basic Medical, Shanghai Medical College, Fudan University, Shanghai, 200032 China

**Keywords:** miR-203, Prostate cancer, Rap1A, Cell proliferation, Cell adhesion, Cell invasion

## Abstract

**Objective:**

Evidence supports an important role for miR-203 in the regulation of the proliferation, migration and invasion of prostate cancer (PCa) cells. However, the exact mechanisms of miR-203 in PCa are not entirely clear.

**Methods:**

We examined the expression of miR-203 in prostate cancer tissues, adjacent normal tissues, PCa cell lines and normal prostate epithelial cells by qRT-PCR. Then, the effects of miR-203 or Rap1A on proliferation, adhesion and invasion of PCa cells were assayed using CKK-8, adhesion analysis, and transwell invasion assays. Luciferase reporter assay was performed to assess miR-203 binding to Rap1A mRNA. Tumor growth was assessed by subcutaneous inoculation of cells into BALB/c nude mice.

**Results:**

Here, we confirmed that the expression of miR-203 was significantly downregulated in prostate cancer specimens compared with matched adjacent normal prostate specimens. Mechanistic dissection revealed that miR-203 mediated cell proliferation, adhesion and invasion in vitro, and tumor growth in vivo, as evidenced by reduced RAC1, p-PAK1, and p-MEK1 expression. In addition, we identified Rap1A as a direct target suppressed by miR-203, and there was an inverse relationship between the expression of miR-203 and Rap1A in PCa. Knockdown of Rap1A phenocopied the effects of miR-203 on PCa cell growth and invasion. Furthermore, Rap1A over-expression in PCa cells partially reversed the effects of miR-203-expression on cell adhesion and invasion.

**Conclusions:**

These findings provide further evidence that a crucial role for miR-203 in inhibiting metastasis of PCa through the suppression of Rap1A expression.

**Electronic supplementary material:**

The online version of this article (doi:10.1186/s13046-015-0125-x) contains supplementary material, which is available to authorized users.

## Introduction

Prostate cancer is the most frequent malignancy among men in most developed countries, and it was estimated to contribute to 28% of newly diagnosed cancers and 11% of cancer-related deaths in 2011 [1]. The progression-free survival rates are so short due to limited treatment strategies, which include surgery, radiation therapy and new therapeutic agents [2,3]. Although the etiology of prostate cancer is still unknown, growing evidence has indicated that multiple changes in specific genes in particular tumors are responsible for the development and progression of PCa [4-7]. There is an urgent need for new, more effective gene therapy programs which can be used in clinical applications.

MicroRNAs (miRNAs) are endogenous small non-coding RNAs of 18–25 nucleotides that act as posttranscriptional regulators of gene expression in diverse biological processes through imperfect base pairing with the 3′-UTR of target mRNAs, inhibiting target gene expression [8,9]. More interestingly, the roles of miRNA have proven to be indispensable in affecting cancer biology, including proliferation, autophagy, apoptosis, and invasiveness [10-14]. Accumulating data have suggested that miRNAs are involved in the tumorigenesis and progression of prostate cancer and act as a tumor suppressors or oncogenes [15-20]. MiR-203, a putative tumor suppressor gene, has been shown to inhibit cell proliferation and invasion and modulate the chemotherapy response in a variety of tumor cells, including lung cancer cells, glioma cells, and breast cancer cells [21-24]. At first, miR-203 has been identified as a skin-specific microRNA, and altering expression of miR-203 in vivo results in promoting epidermal differentiation by restricting proliferative potential and inducing cell-cycle exit through targetting p63, an essential regulator of stem cell maintenance in epithelial stratified tissues [25]. It has been reported that in breast cancer miR203 targets SOCS3 (suppressor of cytokine signaling 3), a negative regulator of fetal liver hematopoiesis and placental development, and ABL1 (Abelson murine leukemia viral oncogene homolog 1), which implicates in processes of cell differentiation, cell division, cell adhesion, and stress response [26,27]. More importantly, miR-203 expression was also observed to be downregulated in prostate cancer tissues, and the over-expression of miR-203 significantly suppresses the growth and invasion of PCa cells [28-30]. Therefore, further exploring the function of miR-203 could broaden the strategies for prostate cancer treatment.

According to the mRNA sequence, Rap1A (Ras-related protein Rap-1A), member of RAS oncogene family, is a predicted target of miR-133a, which shares approximately 50% amino acid identity with the classical RAS proteins and has numerous structural features in common [31]. Researches in leukocytes first demonstrated that Rap1 can enhance cell adhesion and migration and activate survival pathways [32,33]. RAP1 has been indicated to activate the MAPK/ERK pathway, which can contribute cell migration and inhibit cell differentiation [34,35]. In lung cancer, knocking down Rap1A can sensitize cancer cells to chemotherapy [36]. It is also reported that activation of rap1promotes metastasis in prostate cancer and pancreatic cancer [37].

The purpose of the present study was to verify the expression of miR-203 and investigate the molecular mechanisms through which it inhibits tumor growth and metastasis. Our data proved that Rap1A is a direct target of miR-203 and over-expression of Rap1A partly rescue the effect of miR-203 on cell proliferation, adhesion and invasion. All these indicate that miR-203 functions as tumor suppresser through inhibiting Rap1A in PCa and miR-203 may be used in clinical applications for cancer.

## Methods

### Cell lines and cultures

Human prostate cancer cell lines (22Rv1, LNCaP, PC3, DU 145), human HEK293T cells and nonmalignant epithelial prostate cell lines RWPE-1 were purchased from the American Tissue Culture Collection (ATCC) and were cultured in growth medium and under conditions recommended by the supplier.

### Clinical samples

Paired tumorous and adjacent non-tumorous prostate tissues from 53 prostate cancer patients were obtained from the Tongji Hospital Subordinated to Tongji University with prior approval by the Ethics Committee of Tongji University and conducted according to the Declaration of Helsinki. The clinical diagnosis was made by a pathologist and confirmed by hematoxylin and eosin (H&E) staining. Surgical specimens used in this study were radical prostatectomy specimens and selected from the frozen prostate tumor bank if paired frozen blocks enriched for histologically normal and tumor areas were available.

### RNA isolation and quantitative real-time PCR (qRT-PCR)

RNA was extracted with TRIzol reagent (Invitrogen, Carlsbad, CA, USA) from prostate tissues and cells following the manufacturer’s protocol. The quantification of isolated RNA was assessed by a NanoDrop 2000 spectrophotometer (Nanodrop Technology, Inc., Wilmington, DE). Small RNA was converted to complimentary DNA using poly-A polymerase-based PrimeScript™ miRNA qPCR Starter Kit (TaKaRa). Approximately 500 ng total RNA was converted to cDNA using a PrimeScript™ One Step RT-PCR Kit (TaKaRa). Real-time qPCR was performed on the ABI Prism 7900HT (Applied Biosystems, Life Technologies, CA) using SYBR Green (TaKaRa) according to the manufacturer’s instructions. Small nuclear RNA U6 was used as an internal control for miR-203 and GAPDH was used to normalize Rap1A. Primers used for quantification were as follows: (Rap1A forward primer, 5′-GAAGAACGGCCAAGGTTTTGC-3′; Rap1A reverse primers, 5′-CCGTGTCCTTAACCCGTAAAATC-3′; GAPDH forward primer, 5′-GAAGGTGAAGGTCGGAGTC-3′; GAPDH reverse primers, 5′-GAAGATGGTGATGGGATTTC-3′). Each sample was run in three replicates with a dissociation curve analysis.

### Plasmid constructs

To construct the has-miR-203 expression vector, the sequence containing the miR-203 pre-miRNA was amplified by PCR from human genomic DNA using the forward primer 5′-AAATCTAGACCAGGCGAGGGCGTCTAA-3′ and the reverse primer 5′-AAAAGAATTCAGCGGTTCCCACAGCACA-3′. The final PCR product was cloned into the XbaI/EcoRI sites of the pCDH-CMV-EF1-copGFP vector (SBI) according to the manufacturer’s instructions and named pCDH-miR-203.

A 555-bp fragment of the coding sequence of human Rap1A was amplified by RT-PCR using specific primers sense 5′-AAAGGATCCTATGCGTGAGTACAAGCTAGTGGT-3′ and anti-sense 5′-AAAAGAATTCCTAGAGCAGCAGACATGATTTCT-3′ and cloned into pCDNA3.1 (+) vector (Invitrogen) and named it pCDNA- Rap1A.

To construct the firefly luciferase reporter plasmids, the full-length 3′-UTR of Rap1A was subcloned into the pMIR-REPORT™ Luciferase (Lifetechnologies) to generate RAP1A-3′-UTR-WT. A mutant construct of Rap1A 3′-UTR, named RAP1A-3′-UTR-MUT, which carried a substitution of four nucleotides within the predicted miR-203 binding sites of Rap1A 3′-UTR, was carried out using a Phusion® site-directed mutagenesis kit (New England Biolabs). The primers for Rap1A 3′-UTR were 5′-AAAAAGCTTGCCCATAGTCAGCAGCAGCT −3′ and the reverse primer was 5′-AAAACGGCTTACAAGCTGTGGAAGATCTGAAATA −3′.

To knockdown Rap1A in the PC-3 and DU145 cell lines, oligonucleotides targeting Rap1A were designed and annealed to clone into the pLKO.1-TRC cloning vector at the AgeI/EcoRI sites with the forward primer 5′-CCGGCCCAACGATAGAAGATTCCTACTCGAGTAGGAATCTTCTATCGTTGGGTTTTTG-3′ and the reverse primer 5′-AATTCAAAAACCCAACGATAGAAGATTCCTACTCGAGTAGGAATCTTCTATCGTTGGG-3′. The scramble vector (SCR) was used as negative control. All sequences of plasmids were confirmed by DNA sequencing.

### Generation of stable cell lines

The lentiviruses were produced by co-transfecting HEK293T according to the manufacturer’s instructions. For over-expression studies, the lentiviruses expressing miR-203 and control viruses expressing GFP were used to infect PC-3 and DU145 cell lines in the presence of 4 g/ml of polybrene (Sigma). The efficacy of miR-203 over-expression was assessed by qRT-PCR. For Rap1A silencing, PC-3 and DU 145 cell lines were infected as described above for miR-203. Puromycin (4 μg/ml) (Sigma-Aldrich, St. Louis, MO) was added 2 days after infection for the selection of infected cells. Western blots were performed to detect the efficiency of Rap1A in the selected cells.

### Luciferase assays and transient transfections

HEK 293 T cells were co-transfected with 1 μg of RAP1A-3′-UTR-WT or RAP1A-3′-UTR-MUT plasmid and 50 ng of pRL-TK Renilla luciferase expression plasmid with an empty vector control or pCDH-miR-203 using Lipofectamine 2000 (Invitrogen, Carlsbad, CA, USA). Approximately 24 hours after transfection, cells were lysed, and luciferase activity was determined on a scintillation counter with the Dual-Luciferase reporter assay system (Promega). Firefly luciferase activity was normalized to Renilla luciferase activity for each sample. PC-3 and DU 145 cells overexpressed miR-203 were transfected with Anti-miRNA inhibitors Negative Control (anti-NC) and anti-miR-203 inhibitor (anti-miR-203) (Applied Biosystems) at a final concentration of 50 nM with the Lipofectamine 2000 transfection reagent (Invitrogen, Carlsbad, CA, USA) following the manufacturer’s instructions. Each reporter plasmid was transfected at least three times and each sample was assayed in triplicate.

### Cell proliferation assay

Approximately 4 × 10^3^ cells per well in triplicate were seeded in 96-well plates. Cell proliferation assays were assessed at 24, 48 and 72 hours using Cell Counting Kit-8 (CKK-8) (Dojindo, Rockville, MD). The relative number of cells was determined by measuring the optical density of each sample at 450 nm in a microplate reader (Beckman Coulter DTX880 Multimode Detector). All experiments were performed three times, and the average results were calculated.

### Cell adhesion and invasion assay

Approximately 1 × 10^5^ cells were harvested and resuspended in culture medium and then transferred to a 24-well plate that was coated with matrix proteins (40 μg/ml type I collagen or 2 μg/cm2 fibronectin in PBS) at room temperature for 1 h and washed twice with phosphate-buffered saline (PBS) to remove the non-adherent cells. The attached cells were measured by CCK-8 in a microplate reader at 450 nm. The adhesion of cells was related to the control set at 100%.

The cells (2 × 10^5^/well) were suspended in 100 μl of serum-free medium and placed into the upper chamber of the insert with an 8-μm micro-porous membrane (Costar, Corning Inc., MA) coated with Matrigel (BD Bioscience, Bedford, MA). Medium containing 20% fetal bovine serum was added into the lower chamber. Following 24 hours of incubation at 37°C, the cells remaining on the upper surface of the membrane were removed with a cotton swab, and the migrated cells on the lower surface were fixed with methanol and stained with 1% crystal violet for 15 minutes. The stained cells were counted in five randomly selected fields per membrane under an inverted Olympus phase-contrast microscope (100× magnification). Each experiment was performed in triplicate wells and repeated three times.

### Western blot analysis

Total cellular and tissue proteins were lysed in RIPA buffer in the presence of protease inhibitors (Roche Applied Science, Mannheim, Germany). Protein concentration was quantified using the Bio-Rad Protein Assay Kit (Bio-Rad Laboratories, Hercules, CA). Equivalent protein (50 ug) from each sample was loaded, and the lysates were separated by SDS–PAGE electrophoresis and transferred to a PVDF membrane (Bio-Rad Laboratories, Hercules, CA). Membranes were blocked in 5% milk in Tris-buffered saline (TBST) containing 0.5% Tween-20 for 1 hour and incubated with primary antibody (RAP1A, Abcam; RAC1, p-MEK1, p-PAK1, GAPDH, Cell Signaling Technology; MEK1, PAK1, OriGene Technologies) overnight at 4°C. After 3 washes (10 min) in TBST, membranes were detected using horseradish peroxidase-linked goat anti-mouse or goat anti-rabbit IgG antibodies and visualized with the SuperSignal chemiluminescent system (Thermo Scientific, Rockford, IL). The intensity of the bands was analyzed using Bandscan software. All the shown images are from a single experiment that is representative of at least three separate experiments.

### Tumor Xenografts in vivo

Mice were maintained in the animal facility and all experimental procedures using animals were approved by the Institutional Animal Care and Use Committee of Tongji University. DU 145 cells infected with vector or miR-203 at 5 × 10^6^ cells/150 μl were injected subcutaneously into the left flanks of anesthetized 6-week-old NOD-SCID mice. Mice were sacrificed 5 weeks later and tumor weight was determined in grams at the end of the experiment. Tumor samples were excised for western blotting.

### Statistical analysis

All data were presented as the means ± S.D of three or more replications. For relative gene expression, paired Student’s t test was used to determine the statistical significance and two-tailed unpaired Student’s t tests and analysis of variance were used for statistical evaluation of the data. Pearson's correlation analysis was performed to examine the correlation between miR-203 and Rap1A mRNA levels of human PCa tissues. Data analysis was performed using GraphPad Prism 4.0 (Graph Pad Software, La Jolla, CA) and two-tailed p values < 0.05 were considered to be significant.

## Results

### MiR-203 is aberrantly expressed in human prostate cancer tissues and cell lines

**Figure 1:**
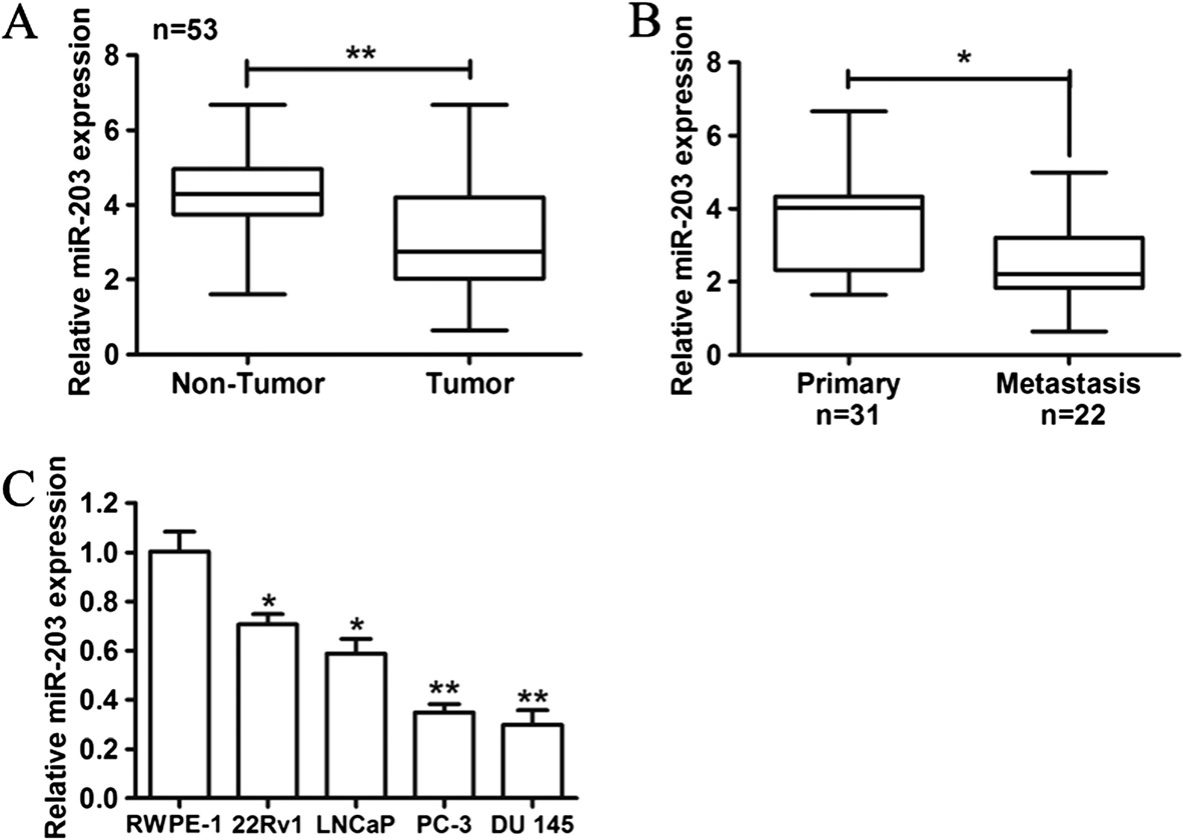
**Expression of miR-203 in PCa samples and cell lines. (A)** The miR-203 expression level was accessed in PCa tissues (n = 53) and matched adjacent normal tissues. **(B)** The miR-203 expression level was determined in metastatic and non-metastatic PCa tissues. **(C)** The relative expression of miR-203 was analyzed in 22Rv1, LNCaP, PC3, DU 145 cells and RWPE-1 cells. The expression level of miR-203 was accessed by qRT-PCR and normalized with an endogenous control (U6). * p < 0.05, ** p < 0.01.

Previous reports have shown that miR-203 was reduced in primary prostate cancer and even more reduced in metastatic PCa. To further confirm the expression of miR-203, especially among Chinese patients, we extended our sample to 53 pairs of clinical specimens. We found that miR-203 was significantly down-regulated in carcinoma tissues compared with the matched adjacent non-tumor tissues (P < 0.01; Figure 1A). Furthermore, in comparison to primary tumors tissues with lymph node and/or bone metastasis, miR-203 levels were significantly lower in PCa tissues without metastasis (P < 0.05; Figure 1B). Similar results were observed in PCa cell lines, that the miR-203 level was significantly decreased in PC-3 and DU 145 cells with high metastatic potential, in comparison to nonmalignant epithelial prostate cell lines RWPE-1 (P < 0.05; Figure 1C). In combination, these results indicated that the down-regulation of miR-203 in PCa may be involved in PCa progression.

### MiR-203 inhibits human PCa cell proliferation, adhesion and invasion

**Figure 2:**
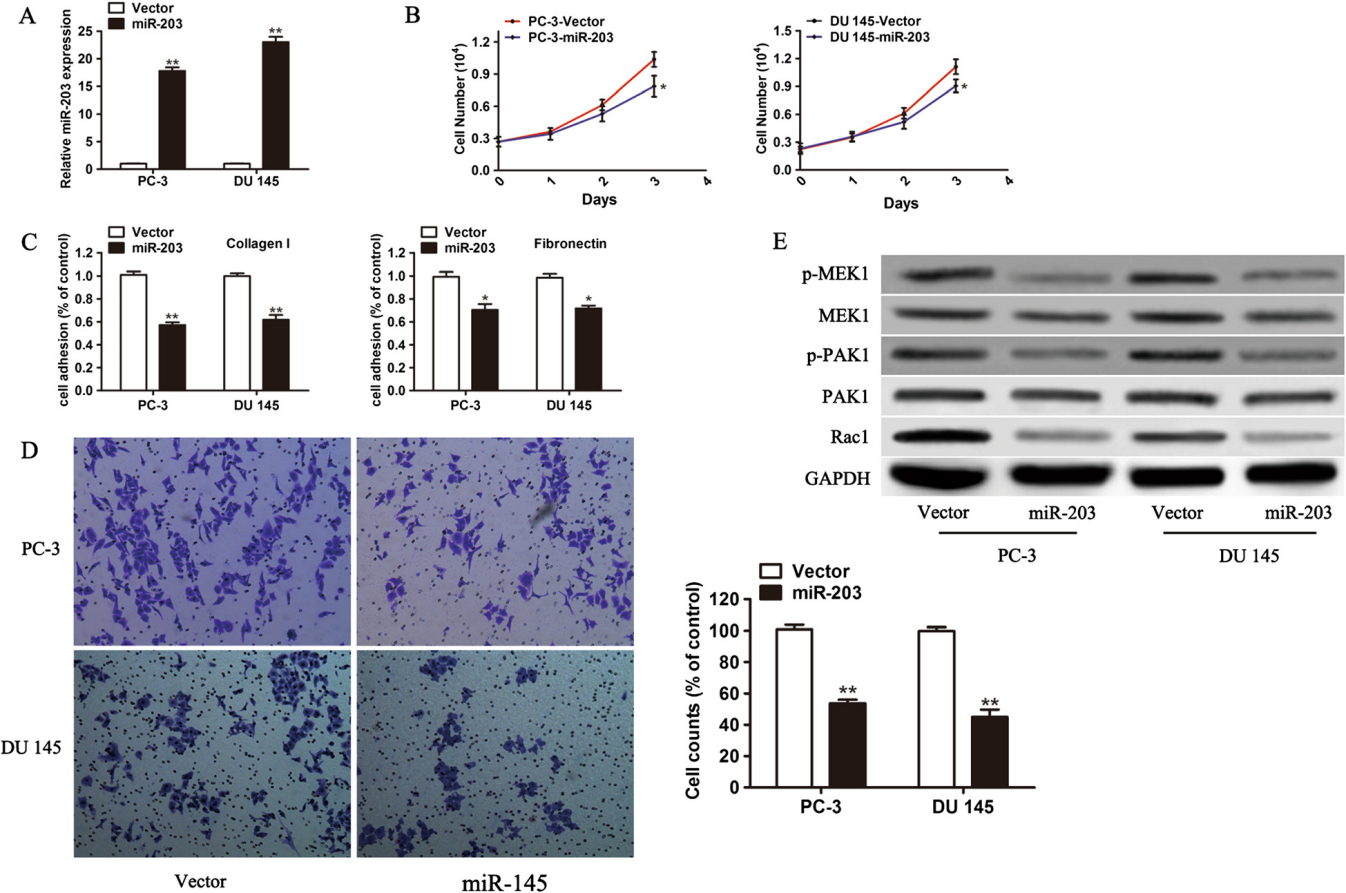
**Effect of miR-203 on PCa cells and regulation of the Rac1/PAK1 pathway. (A)** Relative expression of miR-203 in PC-3 and DU145 cells after infection with miR-203 as determined by qRT-PCR. **(B)** Cell viability was determined by the CCK-8 in both PC-3 and DU145 cells at the indicated time. **(C)** Cell adhesion abilities were assessed in both PC-3 and DU145 cells with or without forced expression of miR-203. **(D)** The migration capacity was assessed in both PC-3 and DU145 cells with or without forced expression of miR-203. **(E)** Western blot analysis shows the expression of RAC-1, PAK1, p-PAK1, MEK1and p-MEK1 after the ectopic expression of miR-203. * p < 0.05, ** p < 0.01.

To investigate the biological functions of miR-203, PC-3 and DU 145 cells with ectopic expression of human miR-203 were generated and the ectopic expression of human miR-203 was verified by qRT-PCR (Figure 2A). To determine whether miR-203 regulates the malignant behavior of PCa, we first studied the effect of miR-203 on the cell proliferation of PC-3 and DU 145 cell lines. Our data demonstrated that the over-expression of miR-203 significantly inhibited relative cell growth (Figure 2B). Cancer metastasis is a multistep cascade, besides tumor growth the adhesion-dependent migration in tissue is another important prerequisite for cancer cell dissemination [38,39]. We then explored any change in cell adhesion and transmigration ability in PC-3 and DU 145 cells over-expressing miR-203. The enforced expression of miR-203 attenuated cell adhesion to collagenase I and fibronectin compared with PC-3 and DU 145 cells infected with empty vector (Figure 2C). Similarly, in the invasion assay, cells infected with miR-203 resulted in a significant decrease in invasion ability (Figure 2D).

To study the molecular mechanisms of miR-203 on cell proliferation, adhesion and invasion, we examined the expression of cell adhesion molecules and downstream pathways. As expected, the over-expression of miR-203 induced a marked reduction in the expression of RAC1, p-PAK1 and p-MEK1 (Figure 2E, Additional file 1: Figure S1), but there is no visible change in the expression levels of total PAK1 and MEK1. These results suggested that the inactivation of Rac-PAK signaling was a key factor.

### Rap1A is up-regulated in PCa, and its expression is inversely correlated with miR-203

**Figure 3:**
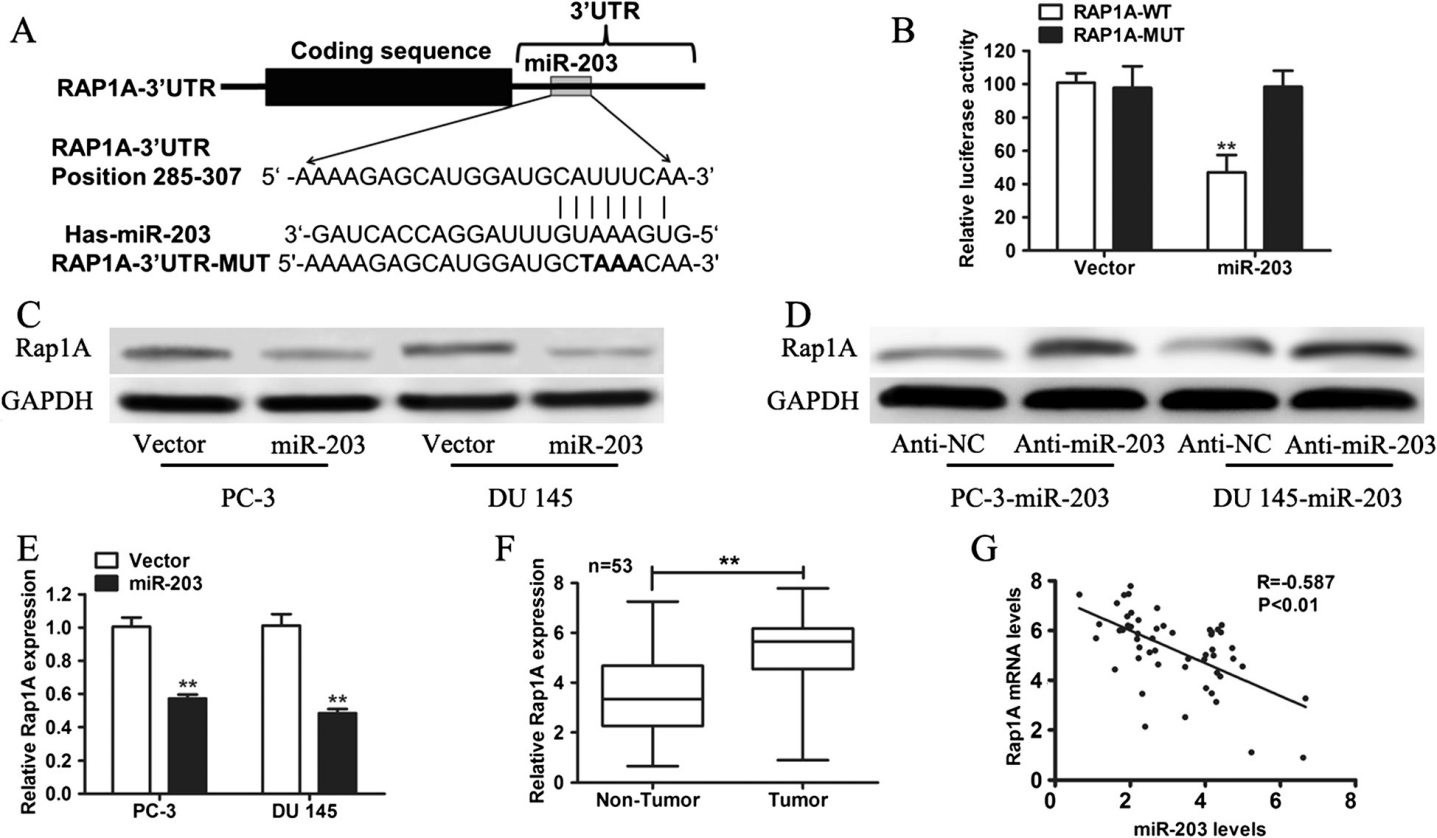
**Rap1A is a new target of miR-203. (A)** The putative miR-203 binding sequence was in the 3′-UTR of Rap1A mRNA. **(B)** A dual luciferase report assay was performed in HEK-293 T cells cotransfected with psi-CHECK2- Rap1A and miR-203. The dual luciferase report assay of HEK-293 T cells cotransfected with RAP1A-3′-UTR-WT or Rap1A-3′-UTR-MUT and empty vector control or pCDH-miR-203. The Renilla luciferase activity was used as control. **(C)** Western blot analysis of the endogenous expressions of Rap1A upon forced expression of miR-203. **(D)** The protein expression of Rap1A in PC-3 and DU145 cells transfected with miR-203 inhibitor or NC was analyzed by western blotting. **(E)** Quantitative real-time PCR analysis showing the mRNA levels of Rap1A. **(F)** The relative expression of Rap1A in PCa tissues and matched adjacent normal tissues was determined by qRT-PCR. **(G)** Plots showing a negative correlation between the relative expression levels of miR-203 and Rap1A in the above samples. * p < 0.05, ** p < 0.01.

To identify the potential target genes of miR-203 that might contribute to its prometastatic function, we used TargetScan (http://www.targetscan.org/) to predict the targets of miR-203 and found that Rap1A was a putative target (Figure 3A). To determine whether Rap1A was a direct target of miR-203, reporter constructs containing full-length Rap1A 3′-UTR were made, along with their corresponding mutant counterpart at the miR-203 target site. Cotransfection of the reporters with miR-203 caused a 50% decrease in luciferase activity compared with the control vector (Figure 3B). Mutating the miRNA binding sites on Rap1A abrogated the miRNA-mediated degradation and rescued the luciferase activity (Figure 3B). These results suggested that Rap1A was a direct target of miR-203. Rap1A protein expression was also significantly decreased in miR-203 over-expressing cells (Figure 3C) and Rap1A protein levels in PC-3 and DU 145 cells with overexpression of miR-203 were increased after transfection with miR-203 inhibitor, compared with a control miRNA inhibitor (Figure 3D). To demonstrate further that Rap1A was negatively regulated by miR-203, we assessed the expression of Rap1A at the mRNA level in the same panel of specimens. The expression levels of Rap1A mRNA were higher in cancer tissues compared with matched adjacent non-tumor tissues (Figure 3E). An inverse correlation between the expression of miR-203 and Rap1A was observed by linear regression analysis. Together, these data demonstrated that that Rap1A is a direct target of miR-203. As Rap1A is reported to function as an oncogene, this may indicate that miR-203 suppresses tumor metastasis through inhibiting Rap1A.

### Effect of knockingdown Rap1A in PCa cells

**Figure 4:**
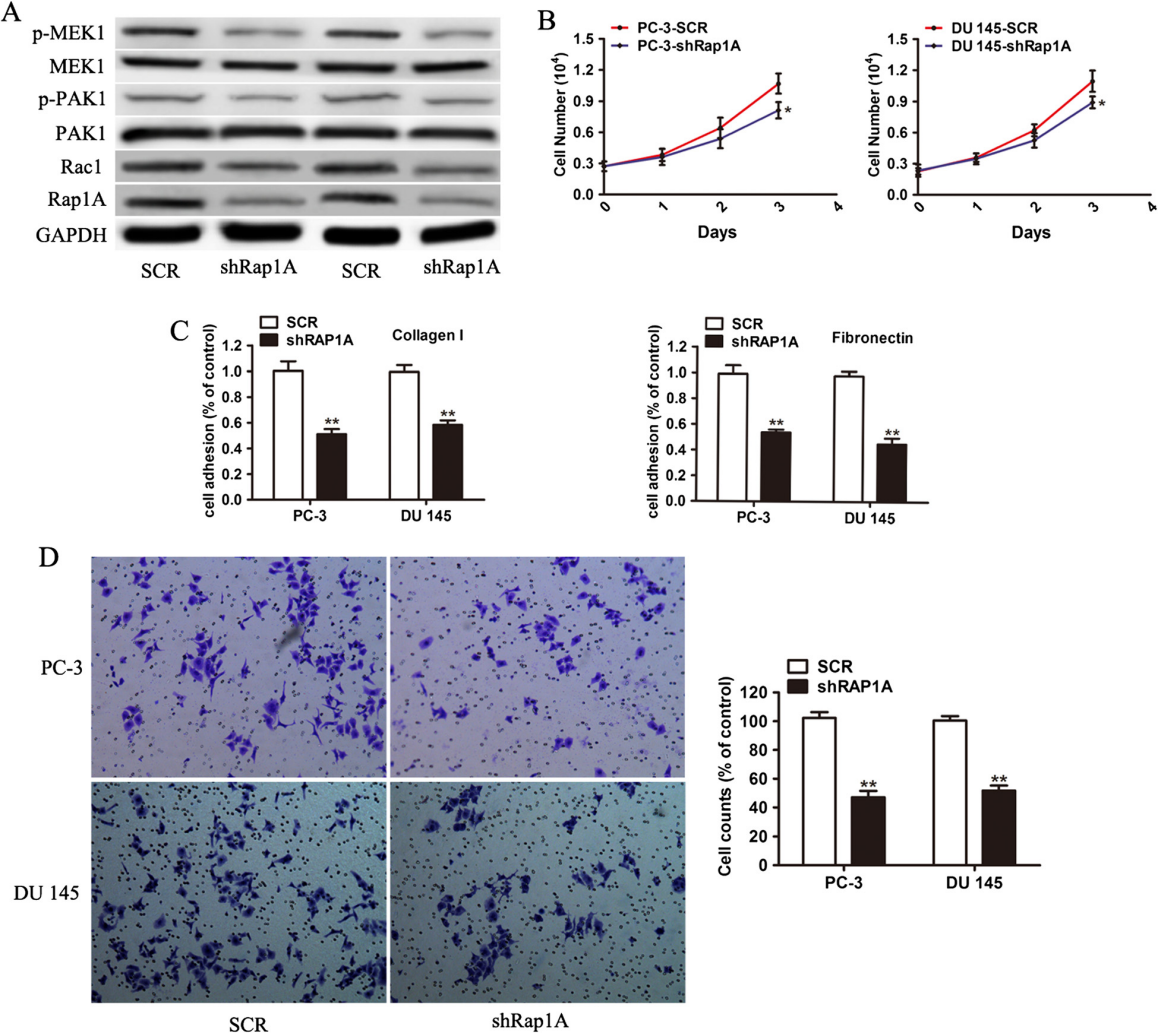
**Knockdown of Rap1A suppresses growth, adhesion and invasion of PCa cells. (A)** PC-3 and DU145 cells were transfected with shRNA for Rap1A (shRap1A) or shRNA-negative controls. The Rap1A protein was analyzed by Western blotting. Cell growth **(B)**, cell adhesion **(C)**, and invasion assays **(D)** were performed with Rap1A silencing. * p < 0.05, ** p < 0.01.

To validate whether miR-203 exerts its tumor-suppressive function through Rap1A, we knocked down Rap1A by RNA interference techniques. The relative Rap1A protein expression levels were significantly decreased after Rap1A knockdown (Figure 4A). Cell viability was significantly inhibited both in sh-Rap1A-transfected PC-3 and DU 145 cells (Figure 4B). Adhesion and invasion assays by various extracellular matrix and matrigel invasion chambers also showed decreased adhesion and invasion activity (Figure 4C and D). To assess the involvement of Rap1A on RAC1-dependent signaling, we measured rac1, p-PAK1 and p-MEK1 levels in PC-3 and DU 145 cells after Rap1A silencing. Consistent with the results of miR-203, the protein levels of rac1, p-PAK1 and p-MEK1 were dramatically reduced (Figure 4A, Additional file 2: Figure S2). Taken together, these results supported that Rap1A was a functional target of miR-203 in PCa.

### Over-expression of Rap1A could partly rescue the effects of ectopic miR-203 expression in PCa cells

**Figure 5:**
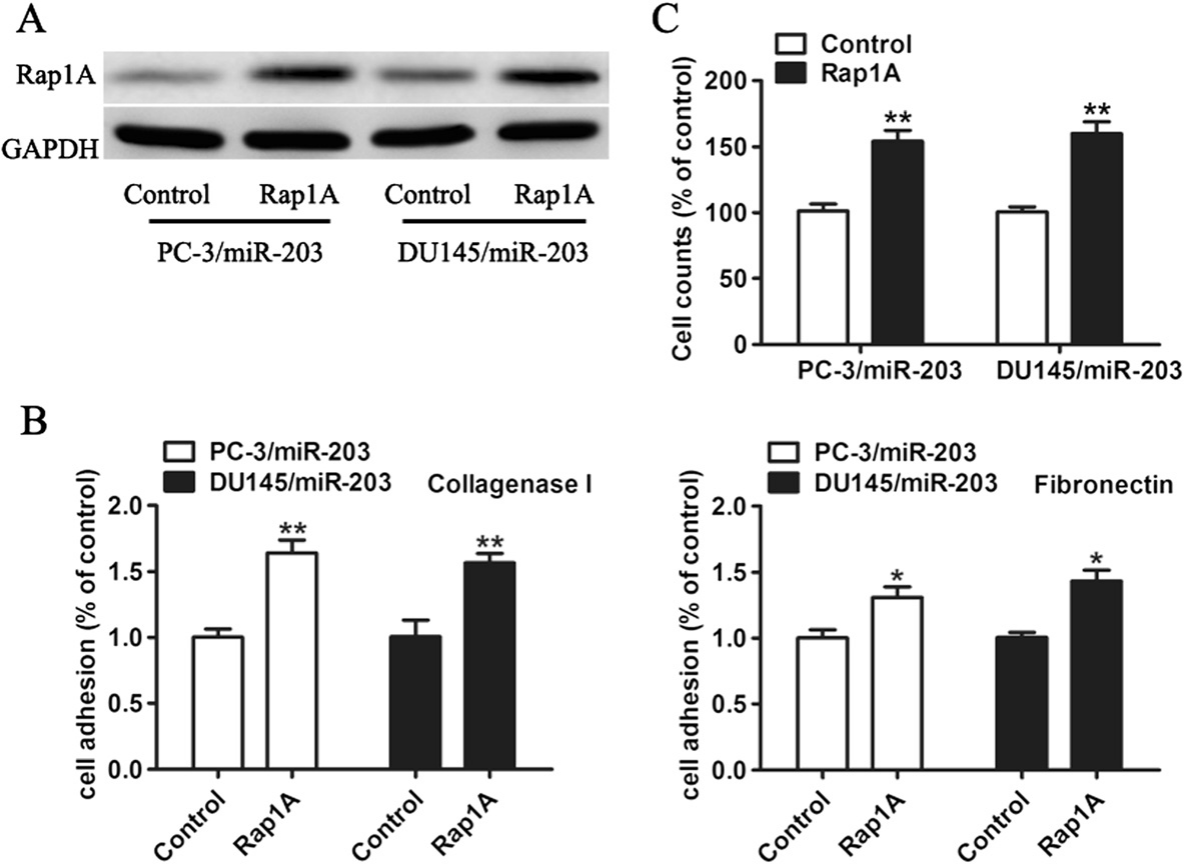
**Rap1A partly rescues migration and invasion inhibited by miR-203. (A)** Western Blot analysis of Rap1A protein expression in PC-3 and DU 145 cells stably expressing miR-203 transfected with control vector or pCDNA-Rap1A. **(B)** The cell viability and the proliferation potential of these cells were detected by CCK-8. **(C)** Transwell assays were performed to evaluate the invasion potential of these cells. * p < 0.05, ** p < 0.01.

To further confirm a functional connection between Rap1A and miR-203, we transfected the control vector or a Rap1A expression vector into PC-3 and DU 145 cells stably expressing miR-203. As shown in Figure 5A, the protein expression levels of Rap1A were significantly increased 48 hours after transfection. We found that expression of Rap1A partly rescue adhesion and invasiveness enhancement of PC-3 and DU 145 cells stably expressing miR-203 (Figure 5B and C), indicating that over-expression of Rap1A could reverse the effects of miR-203 in PCa cells.

### MiR-203 suppresses tumor growth in vivo

**Figure 6:**
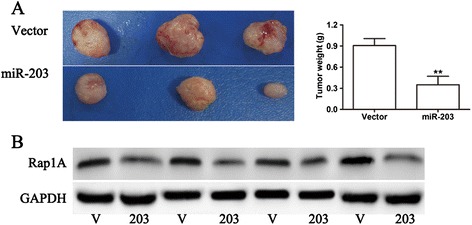
**MiR-203 inhibits tumor growth in vivo. (A)** Representative anatomical photos of xenograft tumors in nude mice injected subcutaneously with DU145 cells infected with vector or miR-203. Tumor weight was measured at the same time. **(B)** Rap1A protein expression in xenografts from vector control groups and miR-203 over-expression groups was assessed by western blot analysis. Lanes 1, 3, 5, 7 were samples from the vector control group. Lanes 2, 4, 6, 8 were samples from the miR-203 over-expression group. ** p < 0.01.

To investigate the contribution of miR-203 in vivo, we injected DU 145cells with miR-203 or control vector subcutaneously into the flanks of nude mice. In this assay, each experimental mouse bearing miR-203-overexpressing and control vector DU 145 cells, respectively, on the right or left dorsal thigh began to exhibit differences in tumor growth between the two sides within two weeks, and the difference continued to increase through the endpoint, at which tumors of the miR-203-overexpressing group displayed a smaller tumor size and weight (Figure 6A). To further explore the in vivo relevance of these observations, we assessed the expression of Rap1A proteins in homogenates from xenograft tumors. Rap1A protein was reduced in DU 145-miR-203 cells compared with control cells (Figure 6B). Taken together, these findings were consistent with the in vitro results and indicated that miR-203 has the ability to suppress PCa cell growth in vivo.

## Discussion

MiRNAs play important roles in a broad range of biological processes including cellular differentiation, proliferation, apoptosis, and tumor development. In the present study, our results provided the first evidence that miR-203 supresses cell proliferation, adhesion and invasion through targeting Rap1A in PCa.

First we verified that the expression of miR-203 was significantly decreased in cancerous tissues of PCa patients compared with non-cancerous prostate specimens. In addition, we found that miR-203 was reduced in PCa cell lines with high metastatic potential compared with the noncancerous prostate epithelial RWPE-1 cells. To further investigate the mechanisms of miR-203 downregulation in PCa, we over-expressed miR-203 in PCa cells and assessed various aspects of PCa cell biology. Introducing miR-203 into PC-3 and DU 145 cells, which express relatively low levels of endogenous miR-203, led to a significant inhibition of cell proliferation, adhesion and invasion. Data indicates that miR-203 functions as tumor suppressor in PCa and is down-regulated in advanced PCa.

Cell adhesion and invasion both play essential roles in tumor growth and metastasis. Then, we focused on the targets of miR-203 which may modulate cellular adhesion and invasion in the present study. Based upon bioinformatics analysis, Rap1A was predicted as a putative miR-203 target and the putative target sites for miR-203 presents within Rap1A 3′-UTR. Rap1 is a member of the Ras family of small GTPases and was activated in response to a number of extracellular stimuli [31]. Many studies have shown that Rap1A was upregulated and involved in cell-cell and cell-extracellular matrix adhesion in many cancers [32-34]. By firefly luciferase activity assay, miR-203 could reduce luciferase activity in the Rap1A wildtype but had no effect on the mutant construct. Meanwhile, the over-expression of miR-203 could lead to a decrease of endogenous Rap1A expression in PCa cells at both the transcriptional and translational levels. Furthermore, miR-203 expression was inversely correlated with Rap1A mRNA expression in PCa tissue specimens. All these results proved Rap1A is a novel direct target of miR-203.

It is complicated to understand the biogenesis of special miRNAs because different direct targets of certain miRNA may play different roles in different organs and different developmental periods. To further validate whether miR-203 plays a tumor-suppressive role by inhibiting Rap1A expression, knockdown Rap1A by shRNA-mediated Rap1A downregulation was confirmed at protein levels. Functional analyses were performed and found Rap1A silencing could phenocopy the effects of miR-203 on phenotypes of PCa cells. Recent reports have shown that the activation of Rap1A was increased in PCa and Rap1A up-regulated the expression of integrins to promote CPa migration and invasion in vitro and in vivo [37]. Here we focused on the roles of Rap1A in cell adhesion and invasion. We have shown that miR-203 over-expression reduced the activation of Rac1/PAK1 pathways. Consistent with this, we also observed inhibition of Rap1A resulted in inactivation of Rac1/PAK1 pathways. Importantly, the significant effects induced by miR-203 over-expression on cell adhesion and invasion were partially reversed by over-expression of Rap1A. All these proved that Rap1A is a functional target of miR-203 and this evidence suggested that miR-203 exerts its tumor-suppressive effects through downregulation of Rap1A in PCa cells. Xenograft assay proved such regulation in vivo too.

Briefly, we found that Rap1A is a novel functional target of miR-203 and both over-expression of miR-203 and knockdown of Rap1A have similar effects in PCa, which is suppressing cell proliferation, adhesion and invasion through inactivation of the Rac1/PAK1 signaling pathway. All data elucidate a new mechanism that miR203 suppresses tumor growth through inhibiting Rap1A. Nevertheless, additional direct target genes mediated by miR-203 remain to be further identified in the future.

## Conclusion

In this study, our data demonstrated a previously unrecognized mechanism that miR203 inhibits cell proliferation, adhesion and invasion through directly targeting Rap1A. MiR-203 could be used in clinical applications for cancer therapy.

## Additional files

Additional file 1: Figure S1. Quantification of mean gray value of each protein normalized to GAPDH. Additional file 2: Figure S2. Quantification of mean gray value of each protein normalized to GAPDH.
