# Supplementary figures and images for: MiR-203 down-regulates Rap1A and suppresses cell proliferation, adhesion and invasion in prostate cancer
Source: J Exp Clin Cancer Res. 2015 Jan 31;34(1):8. doi: 10.1186/s13046-015-0125-x (PMC4321708; doi:10.1186/s13046-015-0125-x)

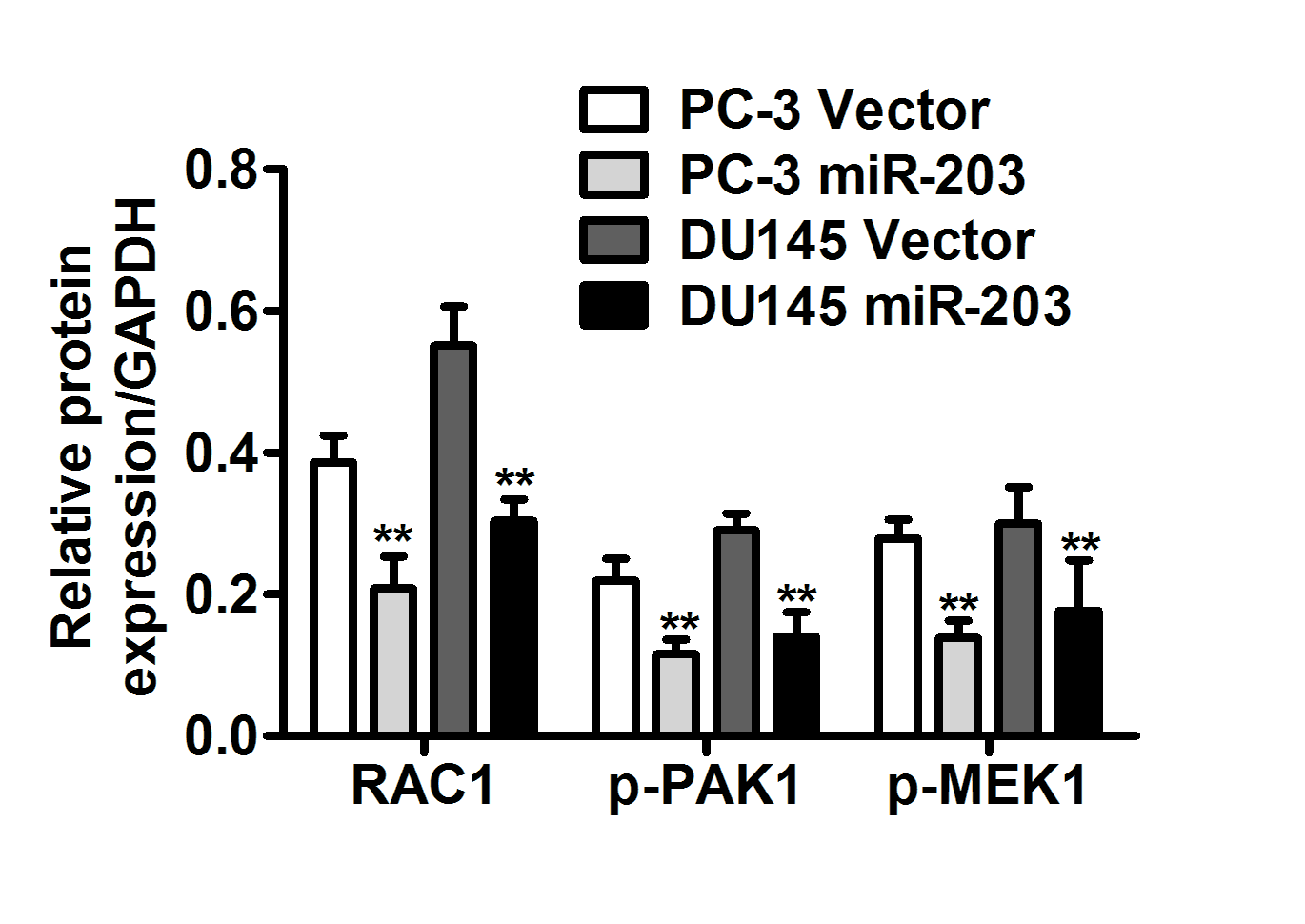

Supplement: Additional file 1: Figure S1. — Quantification of mean gray value of each protein normalized to GAPDH. [file 13046_2015_125_MOESM1_ESM.tiff]

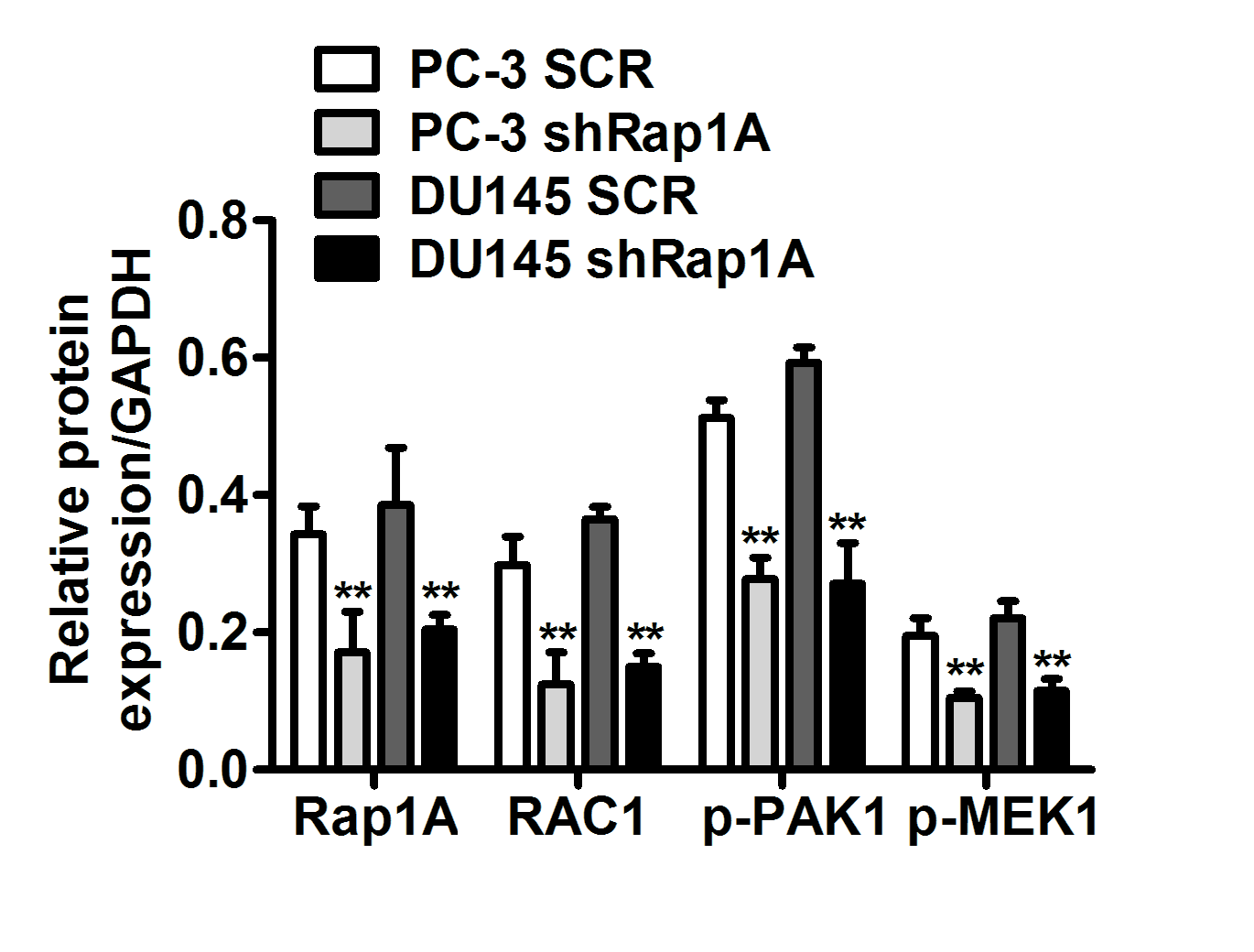

Supplement: Additional file 2: Figure S2. — Quantification of mean gray value of each protein normalized to GAPDH. [file 13046_2015_125_MOESM2_ESM.tiff]
